# Supplementary material for: Implementation and Cross-Validation of a Pharmacokinetic Model for Precision Dosing of Busulfan in Hematopoietic Stem Cell Transplanted Children
Source: Pharmaceutics. 2022 Oct 1;14(10):2107. doi: 10.3390/pharmaceutics14102107 (PMC9611936; doi:10.3390/pharmaceutics14102107)
Supplement: Supplementary file 1 [file pharmaceutics-14-02107-s001.zip › pharmaceutics-1887093-supplementary.pdf]

Paci *et al.* [16]

Model with IOV

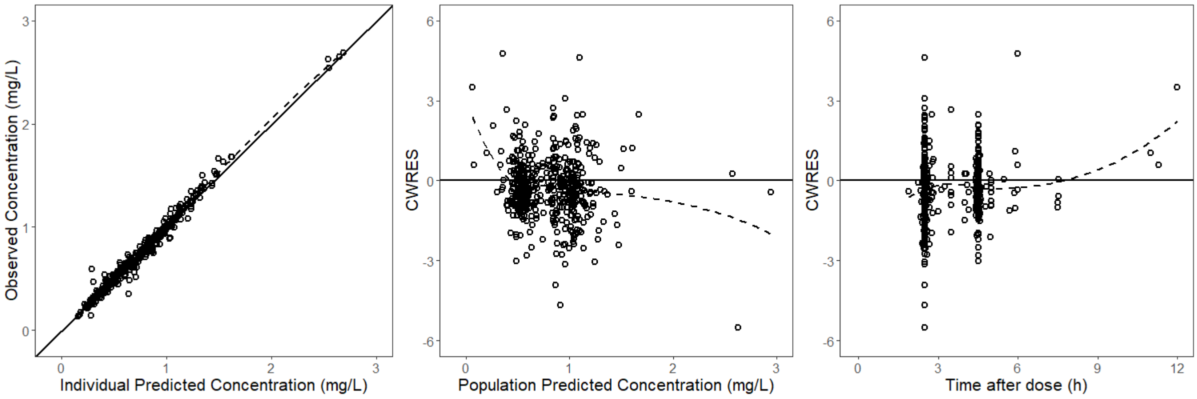

Model without IOV

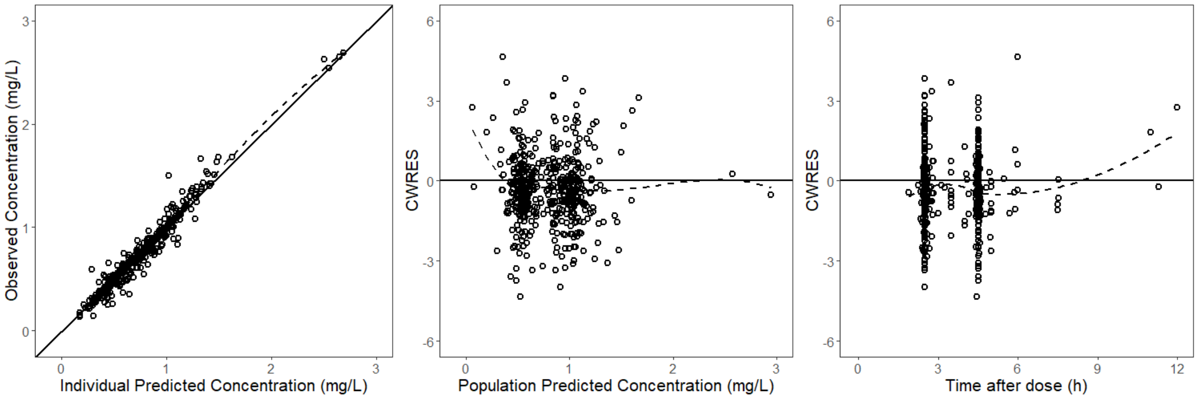

Booth *et al.* [18]

Model with IOV

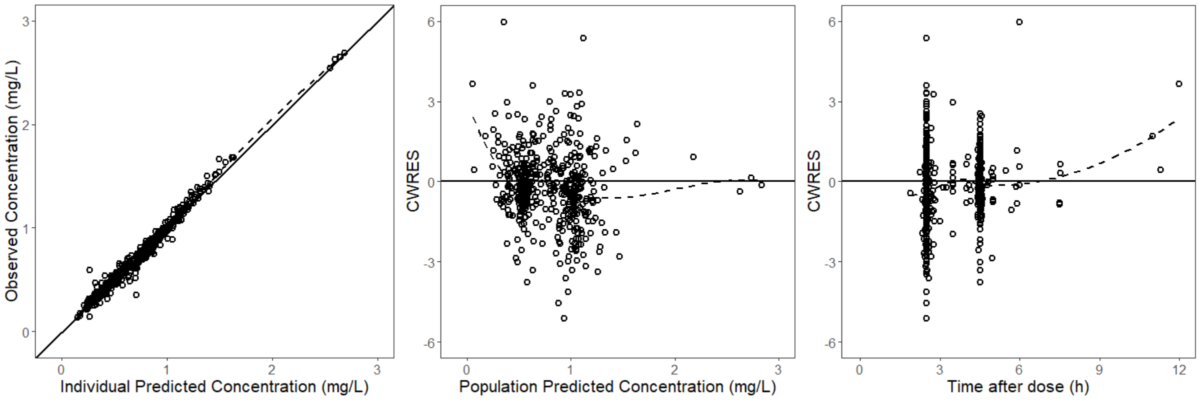

Model without IOV

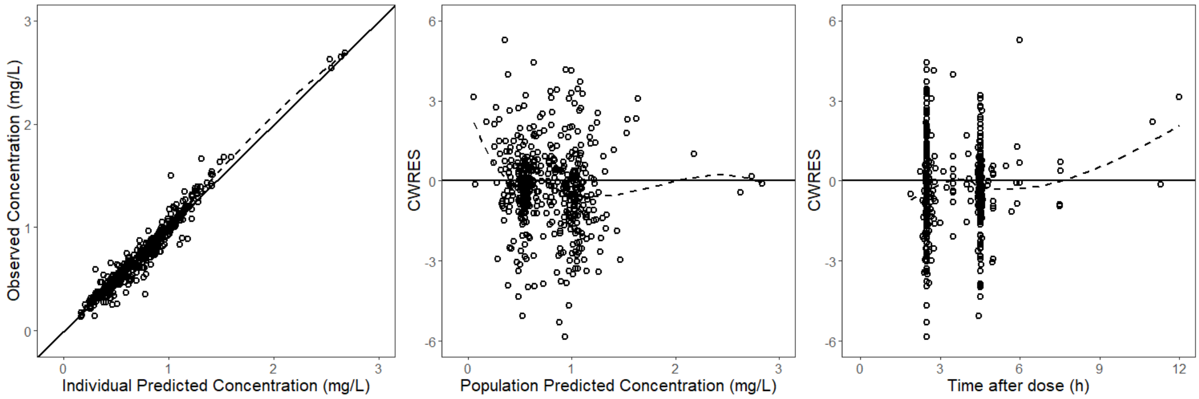

Trame *et al.* [19]

Model with IOV

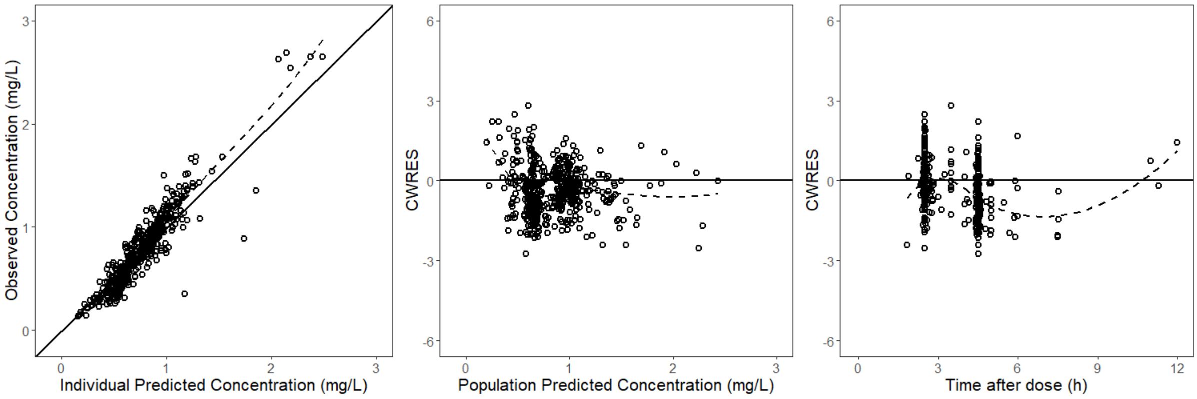

Model without IOV

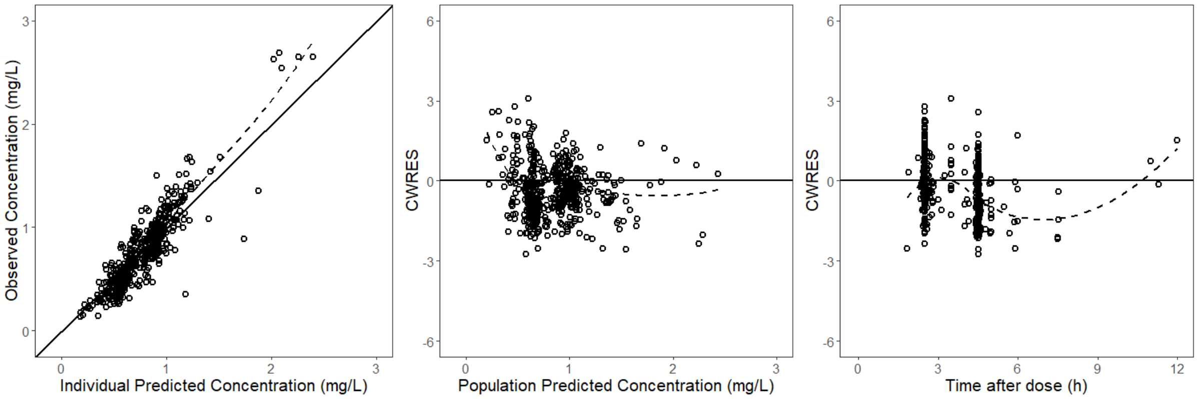

Figure S1: Goodness-of-fit plots of the candidate popPK models with (upper panel) and without (lower panel) interoccasion variability (IOV). Black continuous lines represent identity and ordinate value zero curves as appropriate, dashed lines the loess smooth curves. CWRES, conditional weighted residuals.
